# Noninvasive intracranial pressure assessment using otoacoustic emissions: An application in glaucoma

**DOI:** 10.1371/journal.pone.0204939

**Published:** 2018-10-01

**Authors:** Allison R. Loiselle, Emile de Kleine, Pim van Dijk, Nomdo M. Jansonius

**Affiliations:** 1 Department of Ophthalmology, University of Groningen, University Medical Center Groningen, Groningen, The Netherlands; 2 Graduate School of Medical Sciences (Research School of Behavioural and Cognitive Neurosciences), University of Groningen, Groningen, The Netherlands; 3 Department of Otorhinolaryngology/Head and Neck Surgery, University of Groningen, University Medical Center Groningen, Groningen, The Netherlands; Bascom Palmer Eye Institute, UNITED STATES

## Abstract

The theory that glaucoma patients have a lower intracranial pressure (ICP) than healthy subjects is a controversial one. The aim of this study was to assess ICP noninvasively by determining the relationship between distortion product otoacoustic emission (DPOAE) phase and body position and to compare this relationship between patients with primary open angle glaucoma (POAG), patients with normal tension glaucoma (NTG), and controls. The relationship was also calibrated using published data regarding invasive measurements of ICP versus body position. DPOAEs were measured in 30 controls and 32 glaucoma patients (17 POAG, 15 NTG) at the following body positions (assuming 90° as upright): 45, 30, 20, 10, 0 (supine), -10, and -20°. DPOAE phase had a clear, nonlinear relationship with body position. The mean DPOAE phase shifts between the two most extreme body positions (45 to -20°) were 73.6, 80.7, and 66.3° for healthy, POAG, and NTG, respectively (P = 0.73), and the groups showed the same, nonlinear behaviour. This indicates that there is no evidence that glaucoma patients have a reduced ICP. When calibrated with invasive data, ICP and DPOAE phase were linearly related over an ICP of 3 mmHg. This suggests that, more broadly, DPOAEs could be used in the future to monitor changes in ICP in a clinical setting and to measure dynamic changes in ICP such as diurnal fluctuations or changes induced by certain medications.

## Introduction

Primary open angle glaucoma (POAG) is a chronic and progressive eye disease characterized by loss of retinal ganglion cells, thinning of the retinal nerve fiber layer, and subsequent visual field loss. If left untreated, it can eventually lead to blindness. Previously, an elevated intraocular pressure (IOP) was deemed to be the key factor in the pathophysiology of glaucoma. However, there is a variant of POAG in which the patients have a normal IOP (normal tension glaucoma [NTG])[1]. One potential explanation for this is that the relationship between IOP and intracranial pressure (ICP) is the key factor, rather than IOP itself [2,3].

Alterations in IOP, ICP, or both can lead to a change in the pressure gradient across the lamina cribrosa (LC)—known as the trans lamina cribrosa pressure difference (TLCPD)—and cause it to bulge, therefore damaging the nerve fibers. It has been shown with lumbar punctures (LPs) that patients with glaucoma have a lower ICP than the healthy population and that those with NTG have the lowest [4–6]. As further evidence, patients with normal pressure hydrocephalus who received shunts, which can significantly lower ICP, had a 40 fold increase in the rate of NTG when compared with the general population [7]. There is, however, controversy over this theory [8].

LPs are invasive and thus limit a researcher’s ability to conduct experiments with a sufficient number of subjects. In addition, LP opening pressures are only a proxy of the actual ICP as they are measured at the lower part of the spine and typically only in the lateral decubitus position. They are especially not likely to be representative of the pressure behind the LC. This is important because it is possible that NTG is related to an inability to maintain a certain pressure in the upright position, rather than the lateral decubitus position. Interestingly, a recent study by Linden et al. [8] aimed to use LPs to estimate ICP at the LC as accurately as possible by accounting for the hydrostatic gradients between the auditory meatus and the LC. LPs were done and ICP was measured continuously using a pressure transducer in various body positions but no differences were found in ICP between a small group of NTG patients and controls. A noninvasive device that measures the pressure at the level of the brain rather than in the lower part of the spinal canal could potentially further this field tremendously.

Otoacoustic emissions are sounds that originate in the cochlea and can be easily used to measure cochlear function noninvasively [9,10]. One particular type of emission is the distortion product otoacoustic emission (DPOAE), which is emitted by the inner ear in response to two tones at specified levels and frequencies. These emissions are thought to depend on ICP because there is a connection between the cranium and inner ear via the cochlear and endolymphatic aqueducts. When ICP fluctuates, the pressure on the stapes also changes, affecting the transmission of sound in the middle ear. Previous research has shown that DPOAE phase shifts can accurately represent changes in ICP [11–14]. As such, a DPOAE measurement potentially contains all the desired properties of an ideal ICP measurement. One limitation is that the recorded DPOAE phase has an unknown—subject specific—offset. As a result, if the relationship between ICP and the DPOAE phase would be linear, changes in phase would only convey changes in pressure. In case of a nonlinear relationship (e.g. the phase changes only for pressures above or below a certain value), however, an absolute pressure measurement should be feasible as well. In this study we explore the clinical value of the DPOAE phase shift for ICP assessment and apply the technique to glaucoma patients.

The aim of this study was to (1) determine the relationship between DPOAEs and body position and to (2) compare this relationship between POAG, NTG, and controls. Finally, we aimed to (3) calibrate this relationship using published data regarding LP-based measurements of ICP versus body position.

## Methods

### Study population

Subjects with healthy eyes who responded to our advertisement and glaucoma patients who were selected from the Groningen Longitudinal Glaucoma Study database [15], received an information letter and informed consent form. The ethics board of the University Medical Center Groningen (UMCG) approved the study protocol. All participants provided written informed consent. The study followed the tenets of the Declaration of Helsinki.

In order to be eligible to participate in this study, subjects in all groups had to meet the following inclusion criteria: 50 to 70 years of age and presence of detectable DPOAEs in at least one ear. Additionally, for healthy controls: IOP of 21 mmHg or lower, no eye disease, and no family history of glaucoma (determined by a questionnaire). To exclude eye disease, we performed optical coherence tomography (OCT-HS100; Canon, Tokyo, Japan; considered normal if the mean retinal nerve fiber layer and retinal ganglion cell layer thickness in macular area were above the 5th percentile), frequency doubling technology (FDT; Carl Zeiss, Jena, Germany; no reproducibly abnormal test locations allowed in C20-1 screening mode), and a measurement of visual acuity (visual acuity at least 0.8 in both eyes). For POAG: diagnosed glaucoma and IOP over 21 mmHg before the onset of IOP lowering treatment. For NTG: diagnosed glaucoma and IOP of 21 mmHg or lower before the onset of IOP lowering treatment and at any time during follow-up. Glaucoma was defined according to Heeg et al [15]. We required a reproducible (same hemifield and at least partially overlapping) visual field defect (Humphrey Field Analyzer 30–2 SITA fast; Carl Zeiss, Jena, Germany; criterion: 'glaucoma hemifield test' outside normal limits) in at least one eye that had to be compatible with glaucoma and without any other explanation. Subjects taking acetazolamide were excluded in this study as this drug has been shown to change ICP [16–18].

### DPOAE parameters

DPOAEs were measured using hardware (Elios) and software (Echosoft version 2.4.2) developed by Echodia (St. Beauzire, France). To ensure the highest magnitude responses at the 2*f*1-*f*2 emission, a fixed rate of *f*2/*f*1 = 1.20 with tones at frequencies *f*1 = 1000 Hz and *f*2 = 1200 Hz and levels L1 = L2 = 72 dB SPL were used. All DPOAE measurements were completed in a sound-isolated audiometric room in the otorhinolaryngology clinic.

### Measurement protocol

Blood pressure (Omron Model M6 Comfort, Omron Healthcare Co., Ltd.) and upright IOP (iCare Pro tonometer, Icare Finland Oy) were measured. Subjects were then secured onto the tilt table (Ironman iControl 400 Disk Brake Inversion System, Paradigm Health and Wellness Inc.). Body mass index (BMI) was determined using self-reported height and weight.

DPOAEs were measured at the following body positions (assuming 90° as upright): 45, 30, 20, 10, 0 (supine), -10, and -20°. At each position, 30 seconds were allowed for normalization of the emission, and presumably the ICP [12,19]. This was followed by 5 DPOAE measurements that were performed over approximately 20 seconds. IOP was measured again at the supine position. The ear probe was then removed and subjects were allowed a short break before repeating the test. In this way we were able to determine test-retest variability with (between test) and without (within test) replacing the probe.

### Data analysis

Groups were described with mean and standard deviation (SD) for normally distributed variables; means were compared using one-way analysis of variance (ANOVA). For variables with a skewed distribution, we used median and interquartile range (IQR) for descriptive statistics and the Kruskal-Wallis test for comparing medians of groups. Proportions were compared using a chi-square test.

The stimulus and data collection protocols are already described in detail by Avan et al [20]. At each body position, 5 DPOAE measurements were taken, which took approximately a total of 20 seconds of measurement time. For the within test test-retest variability, the first 2 and last 2 of these 5 measurements were averaged for each body position and compared. For the between test test-retest variability, the average phase over the full 20 seconds was compared for the first and second test, between which the ear probe had been removed and replaced. Test-retest variability was presented as the SD of differences of both measurements.

Due to the subject specific offset of the DPOAEs, normalization of the data was required. For each subject, an average phase over all body positions was calculated and the individuals’ data were normalized using this value. Individual measurements with a signal to noise ratio (SNR) of less than 3 dB were excluded. If fewer than 2 measurements with a SNR over 3 dB were available at more than one body position (out of 5 measurements per body position), those subjects were removed entirely. All analyses were performed using R (version 3.0.2; R Foundation for Statistical Computing, Vienna, Austria). A p-value of 0.05 or less was considered statistically significant.

## Results

Of the 35 healthy subjects who agreed to participate, 1 was excluded because of an abnormal eye exam and 1 because of lack of emissions. Of the 43 glaucoma patients who agreed to participate, 7 were excluded because of lack of emissions. Due to signals below the noise floor, we could not analyze the data of 3 of 35 controls, 1 of 22 POAG patients, and 3 of 21 NTG patients. Therefore a total of 62 participants were included in the analysis (30 healthy controls, 17 POAG, and 15 NTG). Table 1 shows the demographics and ocular characteristics of the study population. The groups had approximately the same mean age, did not differ regarding gender, and there were no significant differences in any of the other demographic criteria. The visual field defects were similar in both the better eye and the worse eye for POAG and NTG. IOP (reported as the mean of both eyes) increased more from upright to supine for the patient groups than for the controls, although this was only statistically significant between controls and POAG (p = 0.01).

**Table 1:** Demographics of the study population (n = 62; mean ± SD unless stated otherwise).

Group	Healthy (n = 30)	POAG (n = 17)	NTG (n = 15)	P value
Gender (% female)	43%	35%	60%	0.36
Age (yrs)	58.4 ± 6.4	61.6 ± 4.1	62.1 ± 4.7	0.05
BMI (kg/m^2^)	25.9 ± 3.1	25.7 ± 3.5	24.8 ± 3.9	0.58
SBP (mmHg)	131.4 ± 11.1	139.8 ± 18.9	131.1 ± 14.9	0.13
DBP (mmHg)	83.5 ± 9.6	87.0 ± 9.9	83.2 ± 10.3	0.44
VF MD of better eye (dB; median [IQR])	-	-2.5 (-6.8 to -0.7)	-3.3 (-4.2 to -2.3)	0.56
VF MD of worse eye (dB; median [IQR])	-	-12.3 (-15.5 to -4.9)	-9.8 (-4.7 to -21.8)	0.74
IOP0 (mmHg; median [IQR])	-	30.0 (28.0 to 34.0)	17.5 (15.2 to 19.7)	-
Upright IOP (mmHg; median [IQR])	15.1 (14.2 to 15.8)	15.6 (14.9 to 16.4)	15.4 (12.7 to 16.0)	0.34
Difference in IOP supine to upright (mmHg; median [IQR])	1.5 (0.9 to 2.0)	2.6 (1.9 to 3.4)	1.9 (0.9 to 3.8)	0.01

*SD* standard deviation, *BMI* body mass index, *SBP* systolic blood pressure, *DBP* diastolic blood pressure, *VF MD* standard automated perimetry mean deviation, *IOP0* intraocular pressure before onset of IOP lowering treatment.

**Fig 1:**
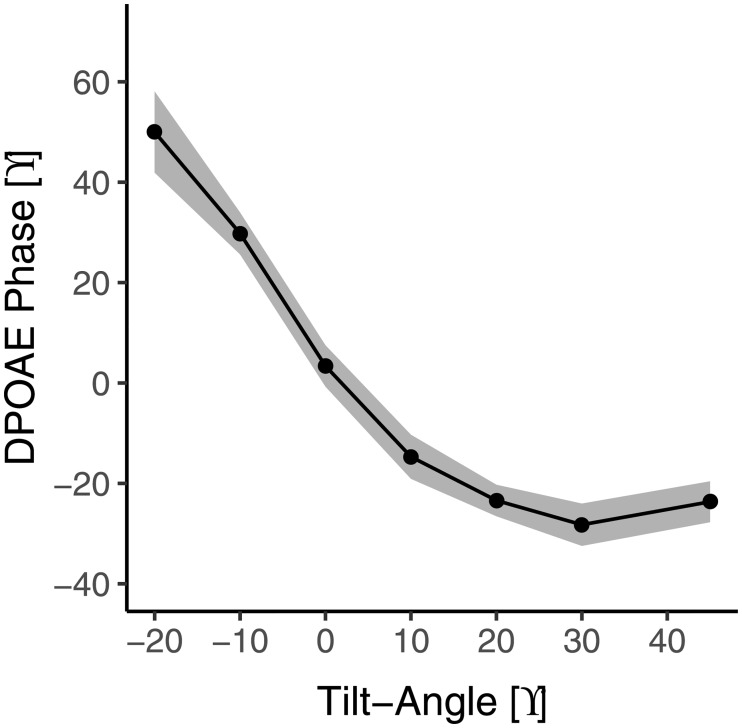
Relationship between body position and DPOAE phase (mean ± standard error) for healthy subjects (n = 30). Data were averaged over both tests. Body positions are in reference to 90° as upright.

Fig 1 shows the DPOAE phase as a function of body position for the 30 healthy subjects, averaged for the first and second test. There is a clear relationship between phase and body position, especially when tilting to the 10° body position and lower. The relationship is not strictly monotonic, as the phase seems to increase again for the 30° body position and higher. This finding is likely robust, as it could be observed in both the first and the second test if analyzed separately. Initially we also measured -30° below horizontal, but that position had to be abandoned due to complaints and discomfort for the participants. In the 12 healthy subjects, 9 POAG, and 2 NTG patients in whom we measured this position (-30°), we found a clear further increase in phase compared to the -20° position of (mean ± standard error) 15.4 ± 3.7, 19.4 ± 9.9, and 24.6 ± 14.8° for healthy, POAG, and NTG, respectively.

**Fig 2:**
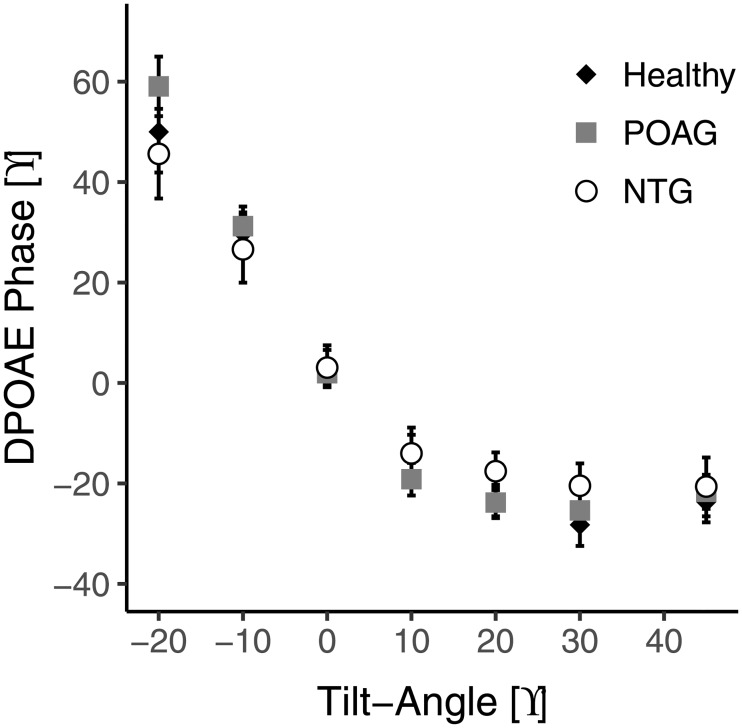
Relationship between body position and DPOAE phase (mean ± standard error) for healthy subjects (n = 30) and POAG (n = 17) and NTG (n = 15) patients. Data were averaged over both tests. Body positions are in reference to 90° as upright.

**Fig 3:**
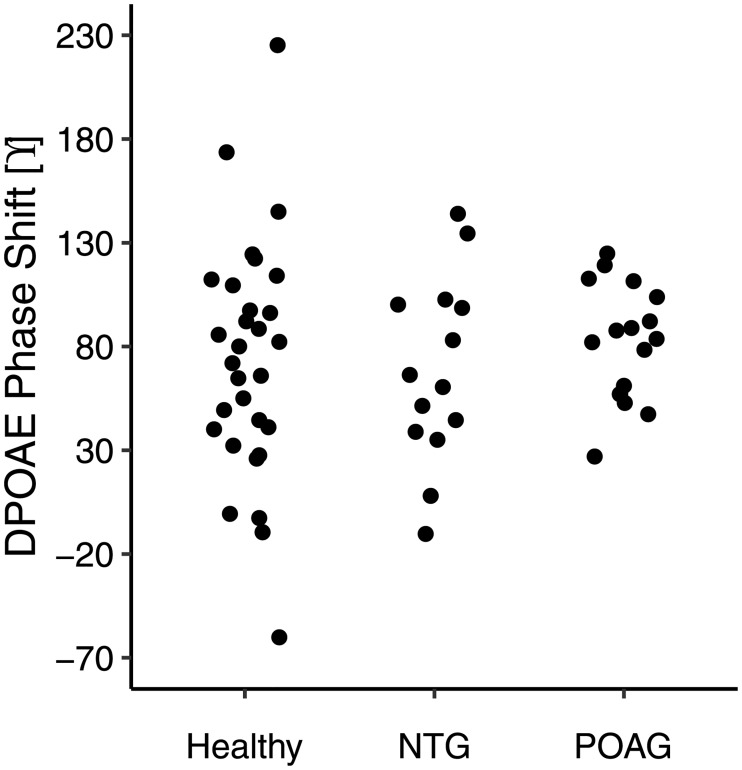
DPOAE phase shifts for healthy subjects (n = 30) and POAG (n = 17) and NTG (n = 14) patients between the body positions of 45° and -20°.

The observed relationship between DPOAE phase and body position was also shown in POAG and NTG (Fig 2). The mean overall phase shifts between the two most extreme body positions (45° to -20°) were 73.6, 80.7, and 66.3° for healthy, POAG, and NTG, respectively. Fig 3 shows the corresponding scatter plots. One NTG subject was not included in this plot because he/she did not feel comfortable tilting to the furthest position. Despite the trend for a smaller mean overall phase shift in NTG patients, an ANOVA for the overall phase shift revealed that there were no significant differences between the healthy subjects and either of the patient groups (p = 0.73).

**Fig 4:**
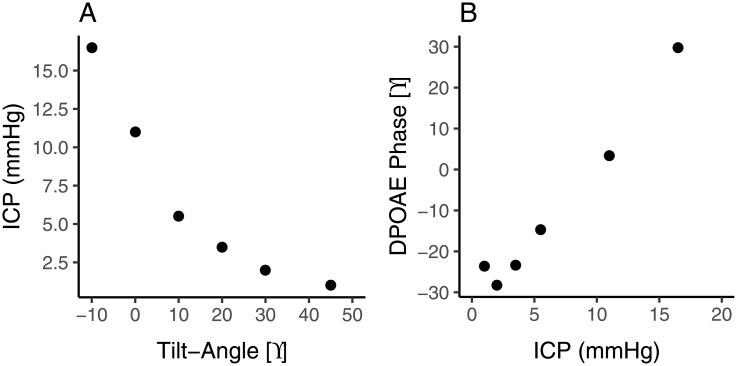
DPOAE phase as a function of ICP. (A) ICP as a function of body position for healthy subjects (n = 11) from Linden et al [8] adapted by interpolation to our body positions. (B) Merged data.

In order to be able to interpret the DPOAE phase data, we merged our data of phase as a function of body position presented in Fig 1 with published data regarding ICP as a function of body position, based on LP measurements by Linden et al. [8]. Fig 4A shows their healthy subject data, adapted by interpolation to our body positions (interpolation was not possible for -20°); Fig 4B presents the merged data, showing DPOAE phase as a function of ICP. Above an ICP of approximately 3 mmHg, phase was linearly related to ICP, with a slope of 4 degree/mmHg. Below 3 mmHg, there was no clear relationship between phase and ICP.

**Fig 5:**
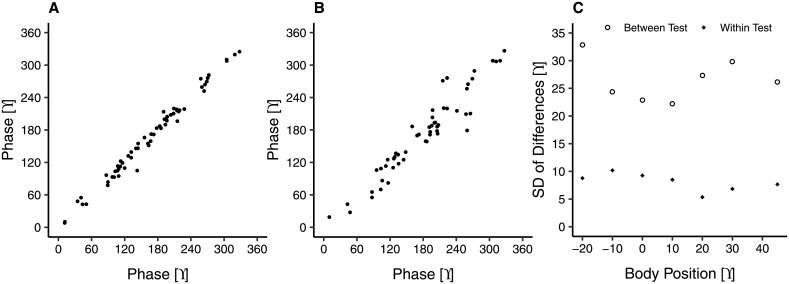
(A) Within test (n = 62) and (B) between test (N = 53) test-retest variability in phase at supine for all subjects and (C) the SD of differences for each body position.

As previously mentioned, the ear probe was removed between the first and second test to examine the test-retest variability of the device. Fig 5 shows scatter plots of the within test (panel A) and between test (panel B) test-retest variability of absolute phase at supine and also the SD of differences at all body positions (panel C). Nine subjects were removed from the between tests comparison, 6 due to a low SNR (for criteria see Methods section) in one of the tests and 3 NTG subjects that were not able to complete the second test due to nausea (see Discussion section). The smallest between test variability, with a SD of differences of 22.2°, occurred at the 10° body position. The within test variability was low and fairly stable across all body positions, with a SD of differences ranging from 5.3° to 10.2° (corresponding to approximately 2 mmHg of ICP; see Fig 4B).

## Discussion

There is a nonlinear relationship between DPOAE phase and body position that is similar in healthy subjects and patients with POAG and NTG. Above an ICP of approximately 3 mmHg, there is a linear relationship between DPOAE phase and ICP with a slope of 4 degree/mmHg; below 3 mmHg, there is no clear relationship between phase and ICP.

Previous studies have demonstrated that DPOAE phase shifts can accurately represent changes in ICP. As one step of a larger experiment, Büki et al. [12] measured DPOAEs in 12 healthy subjects who were tilted on a table between upright and -30° and compared this to data from 5 patients with hydrocephalus and found that “ICP is the key element for all auditory modifications associated with posture”. In another study [13], DPOAEs were measured in 12 healthy subjects titled from upright to -45°. At the same DPOAE frequency as used in the current study, they demonstrated DPOAE phase shifts of about 54° invoked by posture change. The overall shift found in the current study was of the same order of magnitude (~70° for a body position of -20 to +45°). De Kleine et al. [19] provided an equation to calculate changes in ICP from changes in body position. They predicted an ICP change of ~10 mmHg for -10 vs 45°. The DPOAE phase shift for this body position change was approximately 50° in our study. As can be seen in Fig 4, these data are in agreement with each other. In the first study to directly compare DPOAEs with LPs, Bershad et al. [11,21] found that large changes in ICP (>15 mmHg) between opening and closing pressures were significantly associated with changes in DPOAE phase. In another study [14], DPOAEs were measured in 8 subjects undergoing CSF infusion testing, and it was shown that for ICP changes of ~12 mmHg or more over baseline, DPOAE phase changes were significant. In the current study, we have reproduced the finding that changes in DPOAE phase can represent changes in ICP. However, our data suggest that, with appropriate probe placement and sufficient averaging, much smaller changes in ICP could be detected (see below).

For many of the subjects there was a noticeable increase in phase that occurs toward the upright position, so that the minimum phase occurred at 30° and not at 45° (see Figs 1 and 2). There are two physiological possibilities for this phenomenon. One is that ICP has its minimum somewhere halfway between supine and upright rather than at upright. In an invasive ICP study [22] it was demonstrated that this occurred in approximately 5% of subjects. In the current study, however, an observable minimum in phase at body positions lower than 45° occurred in over 50% of subjects. Another possibility is that when there is a negative ICP, as is often the case in the upright position, the stapes is pulled inward into the oval window and may elicit a response in DPOAE phase similar to when it is pushed outward. In this case the minimum phase would occur at a body position intermediate between upright and supine, when the stapes is in a neutral position.

There were some limitations in the current study: First, DPOAEs were not measured at the upright position which was due to a limitation of the tilt table. However, within the range of body positions included in this study, the nonlinear relationship between phase and body position could clearly be uncovered. It is also important to note that 3 NTG subjects could not complete the second part of the test due to nausea and dizziness. No other subjects had any problems with the test. Interestingly, this supports the concept that vascular factors and impairment of ocular blood flow might play a role in the pathogenesis of NTG [23]. Indeed 2 out of the 3 subjects had low blood pressure (106/73 mmHg and 107/79 mmHg). Finally, there was a large intersubject variation in the overall DPOAE phase shift. At first sight, this seems to hamper the use of DPOAE phase for ICP assessment. However, it should be realized that ICP itself also shows a significant variability [22,24].

What information regarding ICP can be obtained from the DPOAE measurements? Importantly, due to a subject-specific offset, there is no one-to-one relationship between DPOAE phase and ICP. For an ICP above 3 mmHg, there is a linear relationship between DPOAE phase and ICP, which implies that a change in phase can be converted into a change in ICP, where 4° corresponds to 1 mmHg (Fig 4B). This suggests that it is possible to monitor ICP changes in a subject or patient in the supine position, in which ICP should be amply above 3 mmHg (Fig 4A). The accuracy (expressed as standard deviation of differences) is approximately 2 mmHg without probe movement, and 5 mmHg with probe movement or replacement. This is already clinically useful; a more stable and reproducible probe positioning could further improve the accuracy. Below 3 mmHg, the phase does not change further, and may even start to change in the opposite direction. This nonlinear behaviour offers potentially an opportunity to obtain information regarding absolute ICP from DPOAE measurements. In the case of a low ICP, one would expect the curve of phase as a function of body position (Fig 1) to move leftward, meaning a more extreme tilt position is required to elicit a DPOAE response. Related to that, lower ICP patients would have a smaller overall phase shift when changing body position from upright to head down. On the other hand, in the case of a high ICP, one would expect the curve to move rightward with a larger overall phase shift.

There are many theories as to why glaucoma may occur in patients with a normal eye pressure. One theory is that NTG patients simply have thin corneas, yielding erroneously low IOP measurements. In this study, however, the corneal thickness for POAG and NTG were similar (544 μm and 554 μm, respectively), signifying that another explanation is necessary. What do our results suggest regarding ICP in NTG? The overall phase shift seemed smaller in the NTG patients than in the healthy subjects or POAG patients (Fig 3), but the difference was not significant. Also, the phase as a function of body position curve of the NTG patients (Fig 2) did not show a clear leftward or rightward shift when compared to that of the healthy subjects or POAG patients. A shift of more than 5° along the body position axis seems unlikely, which suggests that the mean difference in ICP between our NTG patients, healthy subjects, and POAG patients is less than 1–2 mmHg. As such, our results agree with the findings of Linden et al [8], who reported no difference in ICP between NTG patients and healthy subjects, and seem to disagree with Berdahl et al. [4,5] and Ren et al [6], who reported a significant difference in ICP of 3.1 and 3.4 mmHg, respectively between NTG patients and healthy subjects. There is little research on invasive measurement of ICP in glaucoma [4–6,8,25], so it is difficult to determine the cause of the disagreement. One possible reason for the discrepancy proposed by Linden et al [8] is that there is no difference between the studies in regards to ICP in NTG, but there is, however, a difference in the control groups. A normal ICP at supine is considered to be between 7–15 mmHg [24] yet, for the 3 studies that showed a difference between groups, the healthy controls had mean ICPs of ~13 mmHg which is at the higher end of the normal range. In the study by Ren et al [6] there was a relationship between visual field defects and TLCPD, but in this case the IOP was measured in the upright position while the ICP was measured in the lateral decubitus position. Interestingly, in all studies there was no significant relationship between ICP and severity of visual field defects, suggesting something beyond reduced ICP is responsible for the pathophysiology of NTG. Finally, it could be the case that the role of ICP in glaucoma is limited to a small subgroup of NTG patients, that is, those with an IOP at the lower end of the normal range (~10 mmHg; sometimes referred to as low tension glaucoma). These patients were not included in the current study (median sitting IOP 15.4 mmHg; Table 1), and also not in the study by Linden et al [7], where the mean sitting IOP was 15.1 mmHg. However, the mean IOP in the studies Berdahl et al [3] and Ren et al [5] were not clearly lower (14.3 and 16.1 mmHg, respectively). Of note, Ren et al included Chinese patients, whereas in the current study and in the study by Linden et al the patients were of Caucasian origin. When looking at the supine position for healthy subjects in the current study, the ICP determined by DPOAE phase increases by 5.5 mmHg and yet IOP increases by only 1.5 mmHg. This suggests that a large change in TLCPD in the supine position, e.g., during sleep, may be part of a normal physiological pattern.

In conclusion, we did not find evidence that NTG patients have a reduced ICP. Beyond glaucoma, however, DPOAEs can be used to monitor ICP changes noninvasively, which may further clinical care and the understanding of ICP variation and regulation tremendously. First, however, future research should focus on a robust and reproducible probe placement in order to minimize variability.

## Supporting information

S1 Table Underlying information. This is the underlying information for the current study.
(XLSX)
